# Genome-wide analysis of heavy metal ATPases (*HMA*s) in Poaceae species and their potential role against copper stress in *Triticum aestivum*

**DOI:** 10.1038/s41598-023-32023-7

**Published:** 2023-05-09

**Authors:** Tuba Sharf Batool, Roohi Aslam, Alvina Gul, Rehan Zafar Paracha, Mahnoor Ilyas, Kathryn De Abreu, Faiza Munir, Rabia Amir, Lorraine E. Williams

**Affiliations:** 1 Atta-ur-Rahman School of Applied Biosciences, National University of Sciences and Technology, Islamabad, Pakistan; 2 School of Interdisciplinary Engineering & Sciences, National University of Sciences and Technology, Islamabad, Pakistan; 3 School of Biological Sciences, University of Southampton, Southampton, SO17 1BJ UK

**Keywords:** Bioinformatics, Biotechnology, Cell biology, Computational biology and bioinformatics, Molecular biology, Physiology, Plant sciences, Environmental sciences

## Abstract

Plants require copper for normal growth and development and have evolved an efficient system for copper management based on transport proteins such as P_1B_-ATPases, also known as heavy metal ATPases (*HMA*s). Here, we report *HMAs* in eleven different Poaceae species, including wheat. Furthermore, the possible role of wheat *HMAs* in copper stress was investigated. BlastP searches identified 27 *HMAs* in wheat, and phylogenetic analysis based on the Maximum Likelihood method demonstrated a separation into four distinct clades. Conserved motif analysis, domain identification, gene structure, and transmembrane helices number were also identified for wheat *HMA*s using computational tools. Wheat seedlings grown hydroponically were subjected to elevated copper and demonstrated toxicity symptoms with effects on fresh weight and changes in expression of selected *HMAs TaHMA7, TaHMA8,* and *TaHMA9* were upregulated in response to elevated copper, suggesting a role in wheat copper homeostasis. Further investigations on these heavy metal pumps can provide insight into strategies for enhancing crop heavy metal tolerance in the face of heavy metal pollution.

## Introduction

Industrialization has accelerated in tandem with global economic growth. Heavy metal pollution is one of the harmful consequences of rapid industrialization. Its contamination can damage water and soil resources and impact human health via food chains. These metals not only reduce the action of enzymes in cells but can also interfere with the function of crucial ion transporters, impede plant development, and disturb essential cellular processes such as the photosynthetic electron transport^1^. For instance, many enzyme systems are inhibited by cadmium, which affects the proper operation of organs like the liver and kidneys^2^.

Unlike other heavy metals such as cadmium (Cd^2+^), copper (Cu^2+^), mercury (Hg^2+^) and lead (Pb^2+^), copper does not freely bioaccumulate. Hence its toxicity to humans and other mammals is modest. However, chronic exposure to copper leads to liver toxicity, anemia, and severe neurological defects in humans^3^. Plants are also extremely susceptible to copper toxicity due to increased anthropogenic activities. For instance, the release of industrial wastewater with high concentrations of copper, mining, and application of copper-based fungicides on crops results in the accumulation of copper in soil and irrigation water. Plants are directly in contact with contaminated soil; hence, their toxicity in plants is higher than humans and other mammals. Copper concentrations slightly above optimal physiological concentrations lead to metabolic abnormalities and growth inhibition in plants^4^. Excess copper also inhibits many enzymes and disrupts vital elements involved in pigment production and membrane integrity. Its most significant effect is the inhibition of photosynthetic electron transport, which leads to lipid peroxidation^5,6^. Additionally, excess copper in plants can interfere with fatty acid and protein metabolism, inhibition of nitrogen fixation and respiration processes. It is also essential to consider that copper is an effective inhibitor of vegetative development and generates widespread senescence symptoms such as stunted growth of roots and shoots, chlorosis, necrosis, and leaf discoloration^4^.

Heavy metal tolerance has evolved in plants, as have regulatory mechanisms for ensuring an adequate supply of critical nutrients. Membrane transporters play a vital role in the uptake and transport of heavy metal cations and essential micronutrients during the complex mechanisms of heavy metal toxicity resistance. Several gene families involved in heavy metal transport have been identified and investigated. One such family is the P_1B_-ATPases, also known as Heavy Metal ATPases (HMAs), which belong to the P-type family of transporters. This family can selectively transport essential metal micronutrients such as Zn^2+^, Cu^2+^, and Co^2+^ for plant development and distribute non-essential heavy metal ions like Pb^2+^ and Cd^2+^^7–9^. Characteristic features of P_1B_-type ATPases include the presence of 6–8 transmembrane helices (TMs) for cation translocation, an ATP-binding domain (ATP-BD includes soluble nucleotide-binding domain and phosphorylation domains) and a soluble actuator domain (AD). The interconnections of these three domains are critical in regulating the metal ion transport^10^. In addition, they also possess distinct soluble metal binding domains (MBDs). These domains can bind and interact with specific metal ions, such as Pb^2+^ and Cd^2+^, at the N-terminal and C-terminus^11–13^.

*HMA*s have been split into two phylogenetic subgroups based on substrate specificity: a Cu/Ag subgroup and a Zn/Co/Cd/Pb subgroup^13^. *HMA*s have been investigated at the genome level in several species, including Arabidopsis and rice. The Arabidopsis *HMA* (*AtHMA*) family contains eight members, whereas the rice *HMA* (*OsHMA*s) family has nine members^13,14^. *AtHMA*1–4 and *OsHMA*1–3 are members of the Cu/Ag subgroup, while *AtHMA5–8* and *OsHMA4–9* are members of the Zn/Co/Cd/Pb subgroup^13^. *AtHMA6/PAA1* was the first Arabidopsis cloned *HMA* and is involved in the copper transport framework in the chloroplasts^15–17^. More recently, 31 *HMA*s have been identified in *Brassica napus*^18^, 17 in *Populus trichocarpa*^7^, 11 in *Zea mays* and *Sorghum bicolor*^14^, 36 in *Solanum tuberosum*^19^, 20 in *Glycine max*^20^, 14 in turnip landraces^21^ and 32 in *Triticum aestivum*^22^. This indicates that the number of *HMA*s may vary from species to species. The expression of wheat *TaHMA2* was studied during the cadmium stress^22^. The results proposed that microRNAs play a major role in regulating targeted genes such as *TaHMA2* under cadmium stress. However, the role of wheat *HMA*s under copper stress has not been studied previously. In the present study, we aimed to elucidate the evolutionary relationship among *HMA*s of several Poaceae species based on phylogenetic analysis of *HMA* genes. We also explored segmental duplication events exhibited by *TaHMA* and chromosomal distributions of these genes. Furthermore, the expression of wheat *HMA*s was explored under copper toxicity conditions, investigating their potential roles in copper homeostasis and stress tolerance.

## Results

### Identification of P_1B_-ATPases in different members of the Poaceae family

*Oryza sativa* belongs to the family Poaceae, and a high degree of phenotypic synteny has been observed across the entire family^23^. A search was conducted in the MSU Rice Genome Annotation Project Database using the keyword “*HMA,*” which resulted in 68 different sequences. However, only nine contained conserved motifs and thus were considered further. LOC_Os06g48720 *(OsHMA2)* contained only 156 amino acids, whereas other *HMA*s contained more than 900 amino acids. Therefore, LOC_Os06g48720 *(OsHMA2)* was further analyzed on the Pfam protein database to find conserved characteristic *HMA* domains. No significant similarity was found for conserved domains in LOC_Os06g48720 *(OsHMA2)*. Based on this, we considered this as a partial sequence. Thus, this protein sequence was subjected to a BlastP search on Ensembl Plants, and the full-length protein sequence of accession ID Os06t0700700-02 (1067 amino acids in length) was retrieved.

*Brachypodium distachyon* is more closely related to *T. aestivum* than *O. sativa*^24^ and has been proposed as a model species for genetic and molecular genomics studies in cereal crops and grasses^25^. In the case of *HMA*s, no previous study related to *B. distachyon* has been reported. Therefore, the present study used rice HMAs to identify homologs in *B. distachyon* (*BdHMA1-9*). Then *HMA*s homologs for other Poaceae species were identified via BlastP searches in Ensembl Plants using *BdHMA1-9*. This resulted in identifying 27 *HMAs* with conserved domains (E1–E2_ATPase domain Pfam id: 00122 and hydrolase domain Pfam id: 00702) in *T.aestivum* compared to 32 *HMAs* reported earlier^22^. It is important to mention that attempts were made to retrieve the 32 *HMAs* reported by Zhou et al. (2019) using Ensembl Plants, but no significant match was found. Moreover, the sequence IDs were also obsolete on Uniprot and inactivated on Uniparc databases. Thus, here we report the identification of 9 *HMA*s, hence 27 homologs of these genes in the wheat genome (Additional file S1). Furthermore, we also identified 9 HMAs in *Zea mays, Sorghum bicolor*, *Steria italica,* 8 in *Oryza barthii*, *Oryza brachyantha*, and *Triticum urartu*, and 7 in *Saccharum spontaneum* and *Hordeum vulgare*.

### Multiple sequence alignment

Alignment of all 110 *HMA* protein sequences from different species revealed that they possess DKTGT, HP and CPx/SPC *HMA*s conserved motifs. The TGE motif was present in all *HMA*s except TRIUR3_31446-T1 and TraesCS7B02G337700.1. Similarly, the GDxxNDxP motif was present in all *HMA*s except TraesCS6B02G184800.1, HORVU4Hr1G076330.4 OBART07G08010.1, TRIUR3_18572-T, and in all *HMA*7 sequences, the GDxxNDxP motif was replaced by a GDxxNDxA motif.

### Domains, motifs and gene structure analysis of *T. aestivum HMAs*

**Figure 1:**
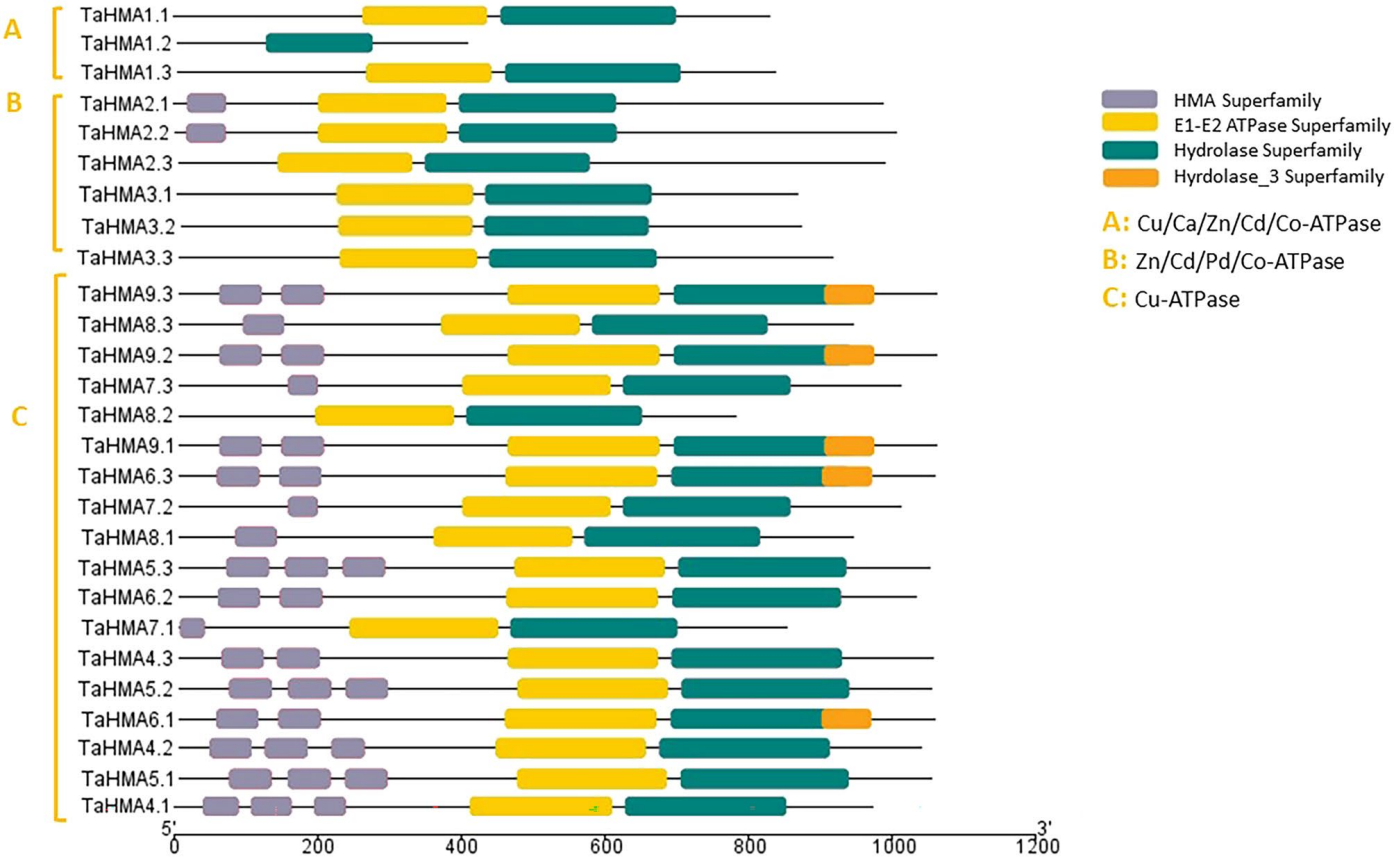
Distribution of conserved protein domains of TaHMA1-27 analyzed using NCBI CDD.

Conserved Domain Database (CDD) analysis of TaHMA1-27 revealed the presence of characteristic domains of HMAs such as hydrolase and E1-E2 ATPase. However, the presence of domains was inconsistent, and some domains were missing in wheat HMAs (Fig. 1). Further analysis of motif diversity in TaHMA1-27 showed the presence of 15 motifs (motif 1–15) within the proteins’ sequences. All TaHMA1-27 contain motifs 1, 10, 11, 13, 14 and 15, whereas a specific motif 9 was present in only clade II (Zn/Cd/Pd/Co-ATPase) and Clade III (Cu-ATPase) (Additional file S2). *TaHMA1-27* length ranged from 3.4 Kbps (*TaHMA3.1*) to 17.78 Kbps (*TaHMA2.1*), their CDS ranged from 2562 to 2958 bps, and protein lengths were 916 aa to 958 aa. Gene structure analysis on GSDS tools showed that *TaHMA1-27* possessed a different number of exons/introns, i.e., 6 exons *(TaHMA3 and TaHMA5)* to 18 (*TaHMA7.2, TaHMA7.3* and *TaHMA8).* However, no regular changes in the number of exons/introns were found in the gene structure of all three clades (Additional file S3).

### Phylogenetic analysis, predicted cellular localization, physiochemical properties and transmembrane helices of poaceae *HMAs*

**Figure 2:**
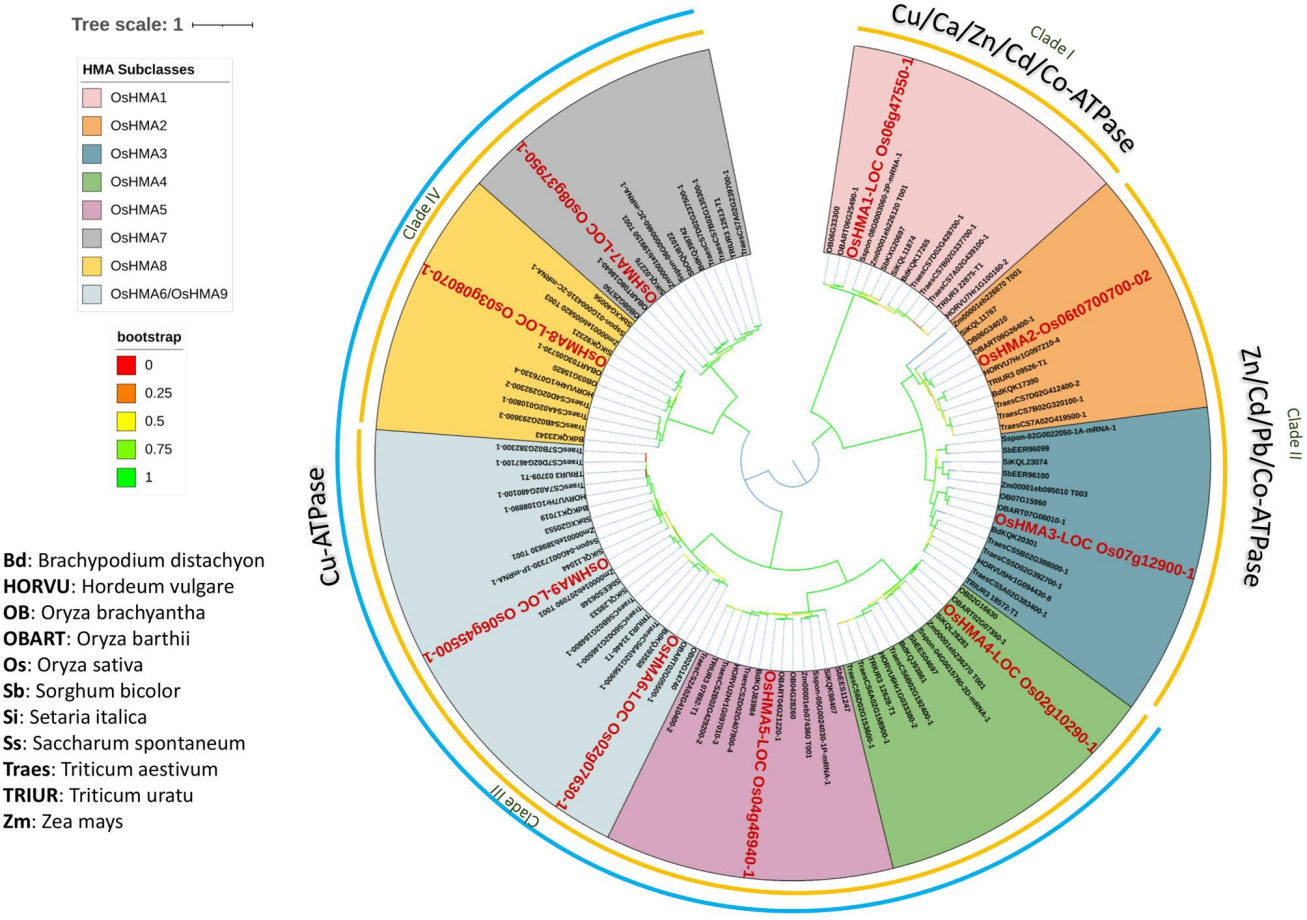
Phylogenetic analysis of P_1B_-type ATPases genes in eleven different hosts of the family Poaceae. Four distinct clades are formed. Clade I comprises Cu/Ca/Zn/Cd/Co-ATPase and contains all HMA1 homologs. Clade II comprises Zn/Cd/Pd/Co-ATPase and contains all HMA2 and HMA3 homologs. Clade III and IV (represented as a blue color arc) comprise Cu-ATPase and contain all HMA4-9 homologs. All HMAs classes are represented with different colors, whereas to distinguish *OsHMAs,* reference sequences are presented with red, bold, and large fonts. Bootstrap values of the phylogenetic tree are represented in different color gradients on the left.

**Figure 3:**
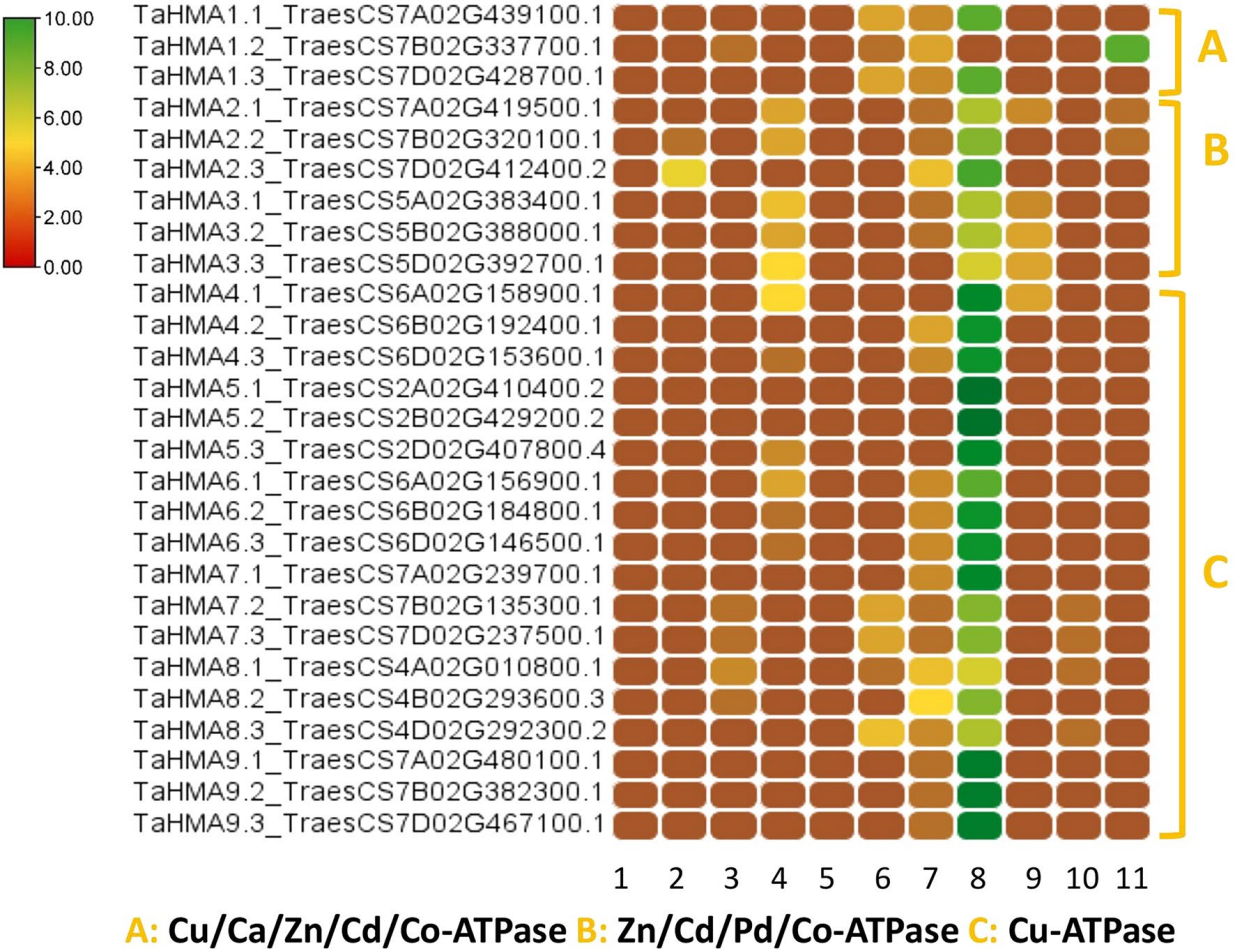
Heat map of predicted localization of each TaHMA1-27 homolog in the cell by WoLF PROST. Map was generated using TB tools software. Here color gradients represent the signals intensity of proteins within the cell. Red means the least expression, whereas green means the greatest expression. 1. Nucleus 2. Cytoplasm 3. Mitochondria 4. Vacuole 5. Cytoskeleton 6. Chloroplast 7. Endoplasmic Reticulum 8. Plasma membrane 9. Golgi bodies 10. Peroxisomes 11. Extracellular protein A: Cu/Ca/Zn/Cd/Co-ATPase B: Zn/Cd/Pd/Co-ATPase C: Cu-ATPase.

The phylogenetic relationship among the HMAs was explored using the maximum likelihood method and 1000 bootstrap replicates among eleven Poaceae species (*B. distachyon, H. vulgare, O. brachyantha, O. barthii, O. sativa, S. bicolor, S. italica, S. spontaneum, T. aestivum, T. urartu, and Z. mays)*. All *HMAs* were characterized into three groups; Group A consisted of Clade I (Cu/Ca/Zn/Cd/Co-ATPase) and contained all HMA1 members; group II consisted of Clade II (Zn/Cd/Pd/Co-ATPase) and contained all members of HMA2 and HMA3*;* group III was comprised of clade III and IV (Cu-ATPase), containing all HMA4-9 members. Notably, within clade IV, HMA6 and HMA9 of all species formed a single cluster showing a close evolutionary relationship (Fig. 2). Predicted physiochemical properties of the TaHMA1-27 showed that the molecular weight of these proteins ranged from 42.548 kDa (TaHMA1.2) to 107.491 kDa (TaHMA5.2), pI ranged from 5.23 (TaHMA6.1) to 7.23 (TaHMA7.2), and GRAVY ranged from -0.162 (TaHMA2.1) to 0.325 (TaHMA6.1). Most TaHMA contained 6–8 transmembrane helices (TMH). However, the number of TMH in eleven out of 27 identified TaHMAs (TaHMA1.1, TaHMA1.2, TaHMA1.3, TaHMA3.2, TaHMA3.3, TaHMA4.1, TaHMA4.2, TaHMA4.3, TaHMA8.1, TaHMA8.2, and TaHMA8.3) were less than six (Additional file S3). The WoLF PSORT prediction algorithm predicted varied cell localization for TaHMAs1-27. These were predicted to localize to the plasma membrane, except TaHMA1.2, an extracellular matrix protein. All TaHMA1-27 members were also predicted to localize to the endoplasmic reticulum except TaHMA3.3, TaHMA4.1, TaHMA5.1, TaHMA5.2, and TaHMA5.3. Furthermore, TaHMA3.3 and TaHMA4.1 were also predicted to show some expression in the vacuole. Only two proteins, TaHMA2.2 and TaHMA2.3, were predicted to localize to the cytoplasm (Fig. 3).

### Chromosomal localization and gene duplication analysis of *TaHMA1-27*

**Figure 4:**
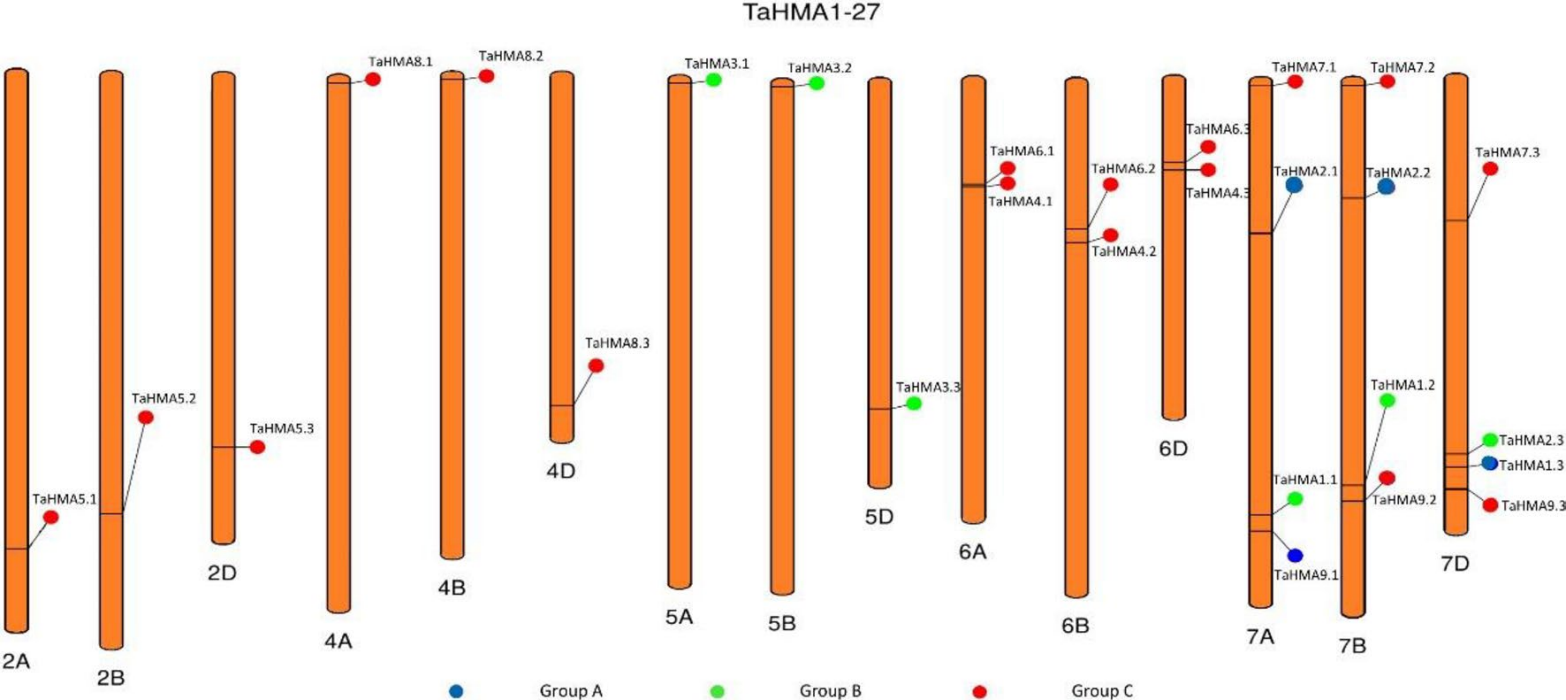
Genomic distribution of *TaHMAs* across sets of ABD genomes. The blue circles represent group A, which is involved in the translocation of Cu/Ca/Zn/Cd/Co ions; green circles represent group B, which involves the translocation of Zn/Cd/Pb/Co ions and red represents group C which is involved in translocation of Cu ions.

**Figure 5:**
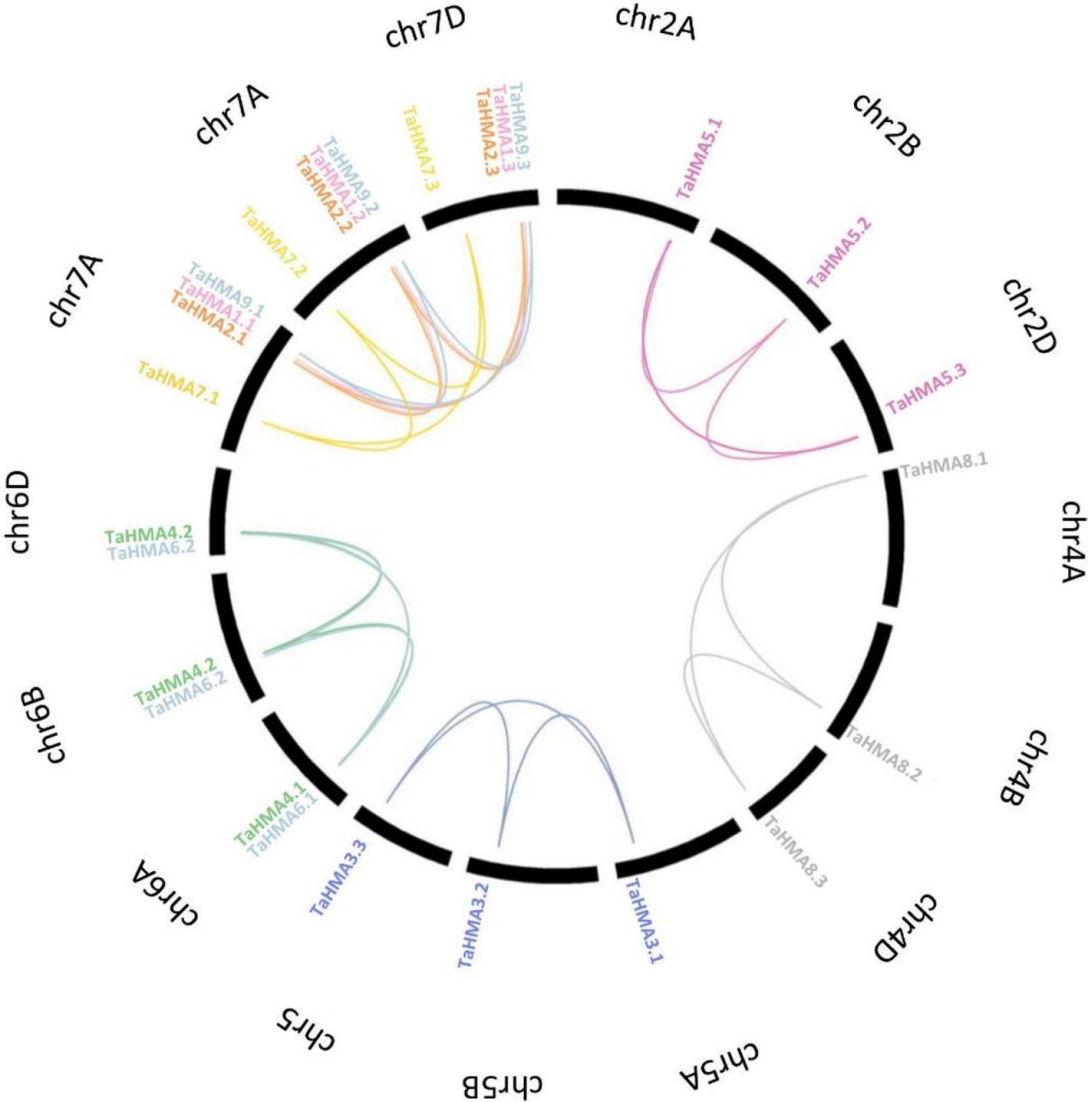
Segmental duplication events exhibited by *TaHMAs*. The 27 segmental gene duplication pairs are connected by different color lines and labeled on 14 wheat chromosomes. Black arc presents wheat chromosomes with chromosome numbers labeled black, whereas names are given in different colors corresponding to the clades of HMAs in Fig. 1.

A PhenoGram plot of the chromosomal localization revealed that *TaHMA1-27* were positioned on 15 chromosomes and distributed on ABD sub-genomes (Fig. 4). Tandem and segmental duplication events are considered important factors of gene family expansion. Analysis revealed that the segmental duplication events related to 15 gene pairs occurred in all studied sub-genomes, whereas no tandem duplication events were found in any of the *TaHMA1-27* homologs (Fig. 5). Furthermore, the ka/ks ratios of the segmentally duplicated *TaHMA1-27* pair ranged from 0.466 to 1.533, with an average value of 0.2895. The ka/ks ratio of *TaHMA5.3/5.2* and *TaHMA5.3/5.1* were > 1, indicating positive selection. However, the ka/ks ratio of the remaining *TaHMA* pairs was less than 1, which suggested that these genes underwent extensive purifying selection^26,27^.

### Phenotypic analysis of *T. aestivum* seedlings grown under copper stress

Wheat seedlings were grown hydroponically on Lombnaes media to investigate the effects of copper toxicity^28^. Two different concentrations of copper were tested (200 µM and 400 µM). Prominent symptoms of copper toxicity chlorosis were observed with stunted growth and reduced weight in copper-treated plants compared to controls. Copper toxicity symptoms such as chlorosis were more pronounced in plants that were exposed to 400 µM as compared to 200 µM copper treatment. Similarly, plants grown under 400 µM copper treatment were more stunted and showed reduced total fresh weight compared to 200 µM copper-treated plants.

**Figure 6:**
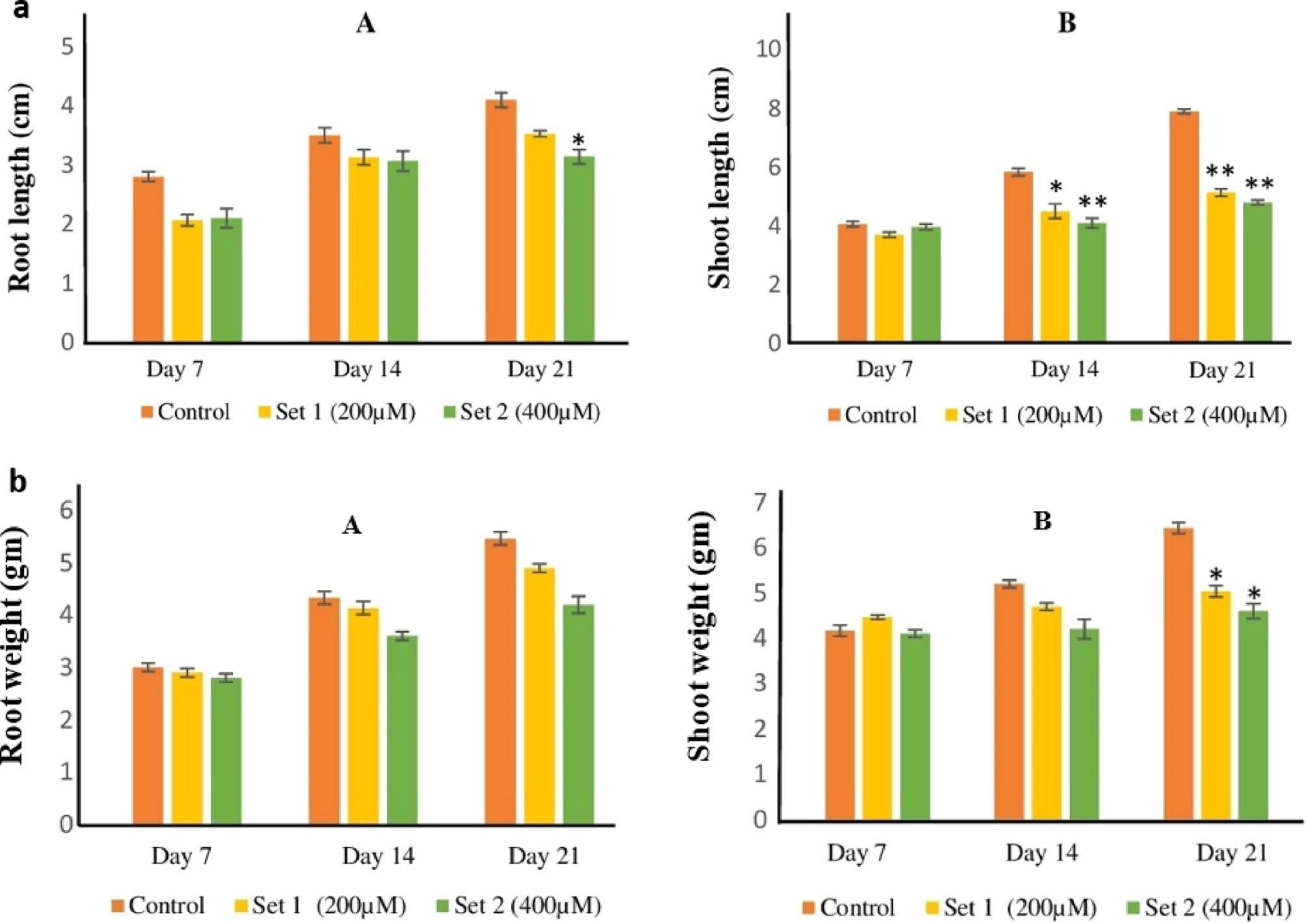
(**a**) Length data of root (**A**) and shoot (**B**) was analyzed by two-way ANOVA with repeated measure design with Bonferroni post-hoc test. *is showing *P-values* < 0.05, ** is showing *P-values* < 0.01 as compared to the control group. Whereas three biological replicates were harvested from each treatment group on day 7, day 14, and day 21 for phenotypic study. (**b**) Fresh weight data of root (**A**) and shoot (**B**) was analyzed by two-way ANOVA with repeated measure design with Bonferroni posthoc test. * shows *P-values* < 0.05 as compared to the control group. At the same time, three biological replicates were harvested from each treatment group on day 7, day 14, and day 21 for phenotypic study.

The data for root length and shoot obtained on day 7, day 14 and day 21 of the set I (200 µM) and set II (400 µM) was analyzed by two-way ANOVA. The statistical analysis shows copper treatment (set II) significantly affected root length on day 21. In contrast, the shoot length (set I) and (set II) was significantly reduced by the treatment on day 14 and day 21 when compared with the control group. Shoot fresh weight data analysis revealed that the treatment in the set I and set II groups for 21 days significantly decreased the shoot length compared to the control group. The fresh root weight data demonstrated a significant interaction between treatment × days. However, post-hoc analysis showed that the mean difference between the groups for fresh root weight did not reach the statistically significant value (Fig. 6a,b). Based on these observations, plants grown under 400 µM copper were further selected for gene expression analysis using qRT-PCR.

### Gene expression analysis in plants grown under copper stress (400 µM)

**Figure 7:**
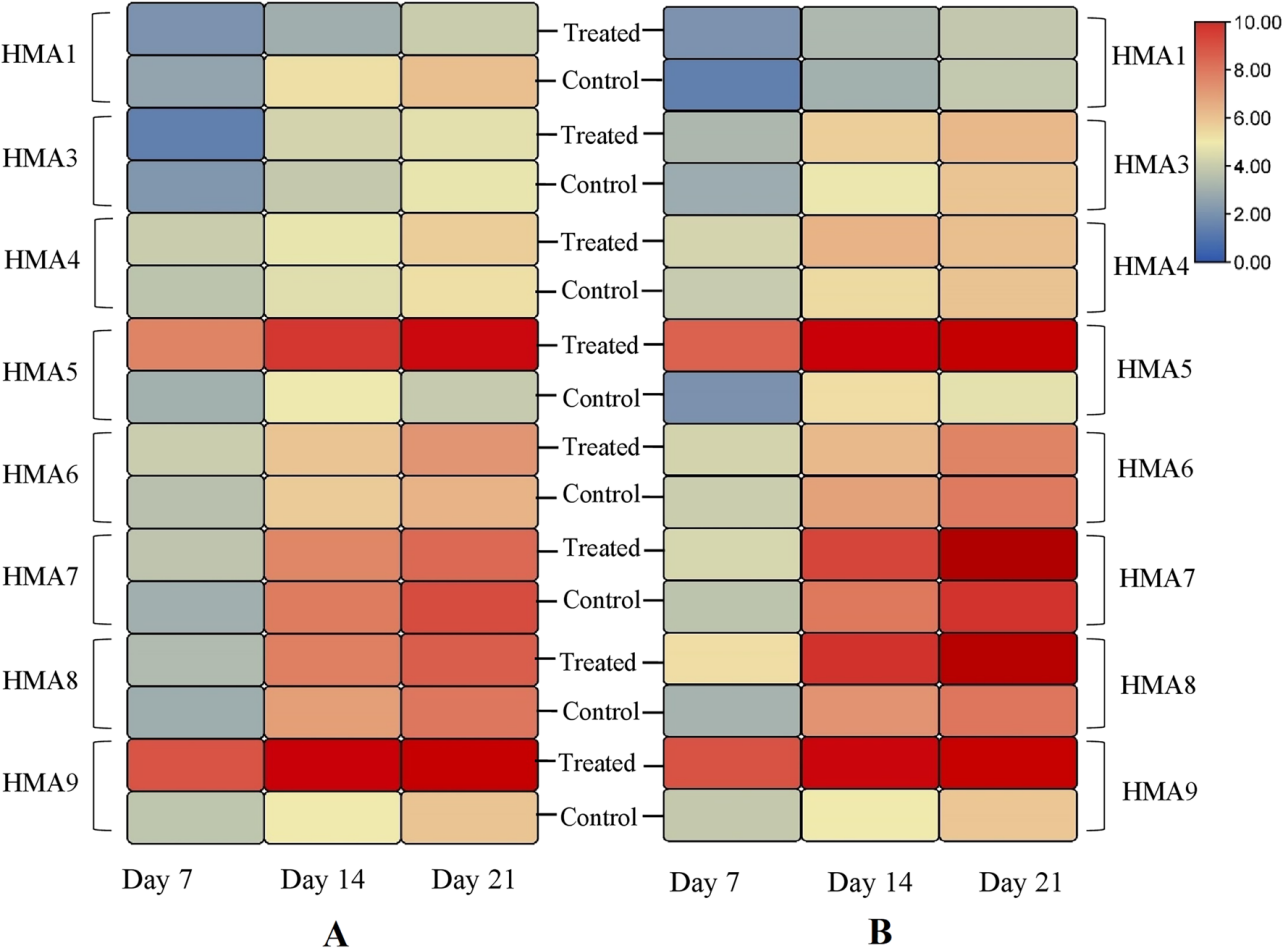
The expression of *T. aestivum HMAs* under elevated copper stress where A = expression of *TaHMAs* in shoots and B = expression of *TaHMAs* in roots. Here color gradients represent signal intensity. Blue means the least expression, whereas red means the greatest expression. Bonferroni test revealed an increase in the expression of *TaHMA5* and *TaHMA9* in both shoot and root on days 7, 14, and 21 (*P-values* < 0.01) in the test group compared to the control samples. Expression of *TaHMA8* was upregulated on days 7, 14 and 21 (*P-values* < 0.01) in roots only. However, in shoots, the expression of *TaHMA8* increased on day 7 (*P-values* < 0.05), 14 (*P-values* < 0.05) and 21 (*P-values* < 0.01). Statistical analysis further showed that expression of *TaHMA7* was increased on day 7 and day 21 (*P-values* < 0.05) in shoots whereas, in roots, *TaHMA7* was upregulated significantly on day 7 (*P-values* < 0.05), 14 (*P-values* < 0.05) and 21 (*P-values* < 0.01) as compared to control.

Statistical analysis of qRT-PCR data shows a significant decrease in the expression of *HMA1* by the treatment in the shoot on days 14 and 21. The *HMA3* expression was significantly downregulated in shoots on day 7 while upregulated in root on day 14. A marked upregulation of *HMA5* was observed in both shoot and root following the treatment on the 7^th^, 14^th^, and 21^st^ day. Compared to the controls, the expression of *HMA6* in the shoot on day 21 and the root on day 14 was significantly upregulated in test groups. In the shoot, the expression of *HMA7* was significantly increased on day 7. However, in the root, the *HMA7* expression gradually increased on days 7 and 14 and drastically increased on days 21. Upregulation of *HMA8* expression in shoot and roots on days 7, 14 and 21 days in test samples was observed. A strong upregulation in the expression of *HMA9* was observed in both shoot and root on days 7, 14, and 21 in the test group compared to the control samples. The analysis did not reveal significant expression differences for *HMA4* in shoot and root. A heat map of the expression pattern of *HMAs* generated from quantitative real-time PCR data under copper stress is represented in Fig. 7.

## Discussion

A range of heavy metals, i.e., copper and zinc, are pivotal to plants due to their roles in various physiological processes. However, when these heavy metals exceed optimal physiological concentrations in plants, these can have detrimental effects. Plants grown on soils contaminated with such heavy metals tend to cope with these stresses by adopting various mechanisms. *HMAs* play a significant role in the trafficking and sequestration of heavy metal field^29^. Still, more information must be given regarding these heavy metal transporters' expression patterns and functioning in wheat under copper stress. In the present study, we identified HMAs in different Poaceae species, conducted phylogenetic analysis and analyzed HMA's specific domains and motifs. Furthermore, chromosomal localization was predicted, and synteny analysis was done to discover possible duplication events in *T. aestivum HMAs*. The expression of *T. aestivum HMAs* under copper toxicity was also determined in *T. aestivum* roots and shoots.

Phylogenetic analysis of Poaceae *HMAs* revealed four clades based on the specification of the ions they translocate. TaHMA1 was in clade I whereas, TaHMA2 and TaHMA3 were in clade II.

The clades III and IV comprised TaHMA4, TaHMA5, TaHMA6, TaHMA7, TaHMA8, and TaHMA9. Previous studies have identified eight *HMAs* in *A. thaliana* and nine *HMAs* in *O. sativa*^13^. Similar heavy metal genes were identified in our study in different Poaceae species, except in *S.spontaneum* and *H. vulgare,* where seven have been identified. In *S.spontaneum* and *H. vulgare,* we initially found nine *HMAs* for both plants*.* However, four were redundant and hence removed from phylogenetic analysis.

There are three primary mechanisms of evolution in gene duplication events: segmental duplication, tandem duplication, and transposition. These elements are mainly responsible for the expansion of the plant gene family^30^. Moreover, plants have maintained various duplicated chromosomal blocks, which results in a high frequency of segment duplication^27^. In this analysis, all identified 27 *HMAs* were located on five out of seven chromosomes across the three sub-genomes (ABD), revealing that the segmental duplication event had participated in the expansion of the TaHMA1-27 homologs. Bread wheat is an allohexaploid (BBAADD genomes, 2n = 6x = 42) that has undergone hybridization events involving three different progenitors of the genera Triticum and Aegilops. The donor of sub-genomes A and B are *Triticum urartu* and *Aegilops speltoides,* derived ~ 7 million years ago from a common ancestor. The first hybridization between sub-genomes A and B resulted in homoploid hybrid speciation of sub-genome D approximately 5.5 million years ago. The second occurred less than 0.8 million years ago, and sub-genomes A and B underwent the event of polyploidization, leading to the formation of the AABB genome. Then the third event, allopolyploidization, gave rise to modern-day wheat less than 0.4 million years ago^31–33^. In the present research, the gene pairs of *TaHMA1-27* homologs were analyzed for an approximate time of segmental duplication using the ka/ks rate. It can be concluded from the calculated Ka/Ks rate that the paralogous pair of *TaHMA1-27* homologs emerged from a recent event of segmental duplication during the formation of the three sub-genomes (ABD) through purification and positive selection^27,34^. Notably, a different number of exons and introns were observed in *TaHMA1-27*. Variations in these exons/introns regions are plausibly due to the consequence of gene fragment integration and realignment. Indeed, this contributes significantly to the evolution of gene families^35^. Previous investigations have revealed that P-type ATPase contains conserved regions DKTGT, PxxK, GDGxNDxP, and S/TGE. In addition, 6–8 transmembrane segments, a CPx/SPC, and an HP locus motif are specific to P_1B_-ATPase and are essential for the metal transport^13,36^. In the present study, all identified *TaHMA1-27* contain conserved hydrolase domain, E1-E2 ATPase domain, DKTGT, HP, CPx/SPC, GDxxNDxP, and TGE motifs. However, the number of TMH in TaHMA1.1, TaHMA1.2, TaHMA1.3, TaHMA3.2, TaHMA3.3, TaHMA4.1, TaHMA4.2, TaHMA4.3, TaHMA8.1, TaHMA8.2 and TaHMA8.3 are found to be less than 6. Therefore, TaHMAs without 6–8 transmembrane domains cannot be assigned as functional HMAs.

In dicots, HMAs are assumed to play a vital part in metal detoxification using efflux mechanisms from the cytosol^13^. Despite their significance, no information is available about the possible role of HMAs in *T. aestivum* in response to copper. Regulation of copper is required for normal growth, development, and normal cellular functionality of plants. However, excessive copper may hamper the availability of other nutrients for plants^37^. In the current research, qPCR was used to investigate the expression of wheat *HMAs* under copper stress. The real-time results analysis showed that the copper stress could increase or decrease the *TaHMA* expression. This revealed that *T. aestivum* exhibits diverse mechanisms of tolerance under copper stress. In the present study, an upregulation of *TaHMA*5 and *TaHMA*9 was found after seven days of copper treatment. The expression of two other *HMA*s, namely, *TaHMA*7 and *TaHMA8,* also significantly increased after 21 days of copper treatment in wheat roots. This indicates that *TaHMA5* and *TaHMA9* may provide a more immediate homeostatic response in wheat following copper exposure.

Most organisms contain P_1B_-type- ATPases, which are key for metal homeostasis. The copper-transporting P-type ATPases are likely to be key players considering their ability for contra electrochemical gradient transport and unique features conserved in plants^11^. In this study, the upregulation of *TaHMA5, TaHMA7*, *TaHMA8* and *TaHMA9* in wheat in response to copper stress suggests their role in regulating copper ions. Previous studies have reported that HMA transporter functions are related to their subcellular localization. For example, studies conducted on Arabidopsis thaliana AtHMA1 are localized at the chloroplast envelope. AtHMA1 is found to be involved in the copper translocation into the chloroplast and detoxification of heavy metal zinc^38,39^.

Similarly, Oryza sativa OsHMA3 and OsHMA4 are localized at the tonoplast and limit the accumulation of copper and cadmium in seeds and roots vacuole^40^^,^^41^. OsHAM9 is a copper efflux protein localized to the plasma membrane. It is expressed in roots and helps in lead, zinc and copper transport^42,43^.OsHMA2 and OsHMA5 are located at the plasma membrane and perform the function of heavy metal transport from root to shoot^44,45^. The earlier investigation also suggested that AtHMA7 is a post-golgi protein that delivers copper to an endoplasmic reticulum-associated protein (ethylene receptors ETR1). These two proteins interact between the sub-compartments of the inner membrane of both organelles^46^. Another study on two ecotypes of *Silene vulgaris* showed upregulation of *SvHMA7* under copper stress, highlighting the role of HMA7 in the cellular copper detoxification^47^. Our analysis of predicted subcellular localization using WoLF PSORT revealed that TaHMA5, TaHMA7, TaHMA8 and TaHMA9 are localized to the plasma membrane, which are the orthologues of OsHMA5, OsHMA7, OsHMA8 and OsHMA9.

Moreover, WoLF PSORT also predicted the subcellular localization of TaHMA7 and TaHMA8 to the endoplasmic reticulum. Therefore, it is predicted that the four TaHMA (TaHMA5, TaHMA7, TaHMA8 and TaHMA9) have similar functions to other reported *HMAs*. However, further experimental studies based on genome engineering are necessary to reveal the molecular mechanism and localization of these TaHMA in response to copper stress.

## Conclusion

This study demonstrated the identification of *HMAs* in 11 different plant species from the Poaceae family and their phylogenetic evolutionary relationship. Identified members were divided into 4 main clades based on the type of metal ions translocation. Three genes, *TaHMA7, TaHMA8,* and *TaHMA9,* were significantly upregulated by elevated levels of copper. Overall, this study indicates that these *HMAs* play an important role in regulating and translocating copper in wheat. Our results will provide a foundation for a better understanding of TaHMA members in wheat for functional characterization.

## Methods

### Cultivation of wheat plants for phenotypic study under copper stress

As reported previously, *Triticum aestivum* seedlings (Var. Sehar-06) were grown hydroponically using the Lombnaes media Field^28^. The use of plants/plant parts/seeds in the present study complies with international, national and/or institutional guidelines. The seedlings were grown under controlled conditions with temperatures at 21 °C/16 °C (day/night) and humidity at 55–65%. The photoperiod was maintained at 16 h at a quantum flux density (PAR) of 220 μmol m^−2^ s^−1^. After 14 days on the control media, three plants (roots and shoots) were harvested, and their fresh weight and length were noted. Plants were then snap-frozen using liquid nitrogen and stored at − 80 °C. The remaining twenty-seven plants were divided into three treatment groups (nine plants in each): control (2 µM CuCl_2_), Set I (200 µM CuCl_2_), and Set II (400 µM CuCl_2_). Plants were grown for 21 days under their respective treatments. Three plants were harvested from each group on day 7, day 14 and day 21 after the start of the treatment, and fresh weight and length of shoots and roots were determined. A two-way ANOVA with repeated measure design followed by Bonferroni post-hoc analysis was used to determine whether there were significant changes between copper treatments. *P-values* < 0.05 were considered significant.

### RNA extraction and cDNA synthesis

RNA was extracted as previously described by^48^. Dnase I (Thermo Fisher Scientific, USA) was used to treat RNA samples to ensure any potential genomic contamination was removed. The first complementary DNA (cDNA) strand was created using ThermoFisher Scientific (USA) cDNA synthesis kit per the manufacturer’s instructions.

### Quantitative real-time PCR (qRT-PCR)

The expression of wheat *HMA*s under copper stress was investigated using *qRT-PCR*. Three replicates were used per treatment per timepoint for qPCR. The primers for wheat *HMA*s were created using Primer3Plus (version 2.4.2)^49^ and validated using Primer-BLAST of NCBI. Actin was used as the reference gene^50^. All the primers utilized in this study are listed in Table 1. The ThermoFisher scientific SYBR Green Kit (Thermofisher scientific, USA) was used to execute the real-time PCR reaction in a 96 well-plate. To prepare a reaction mixture, 2.5 ng cDNA template, 0.3 M of reverse and forward primers, 1X SYBR-green master mix, and sterile 18 MΩ H_2_O (up to 20 µL) were employed. The reaction was run on an Opticon DNA Engine Continuous Fluorescence Detector (Applied Biosystems 7000 Real-time PCR system) under the following conditions: initial denaturation at 95 °C for 2 min, followed by 40 cycles of amplification comprising of denaturation for 50 s at 95 °C, annealing for 5 min at 70 °C and extension for 4 min at 68 °C. The final extension period was carried out for 10 min at 71 °C. The Pfaffl method calculated relative gene expression values^51^. The results were analyzed using two-way ANOVA with repeated measure design followed by Bonferroni post-hoc analysis. Significance was taken at *P-values* < 0.05. Finally, a gene expression heat map was generated using TB tools software^52^.

**Table 1:** Sequence of the primers used in the qRT-PCR study.

Gene name	Forward primer	Reverse primer
*TaHMA1*	TTGGTTGACGGTTCTTCTCC	TCAACTTTGCCGAGACGTAGT
*TaHMA3*	CTGCCACAAGCCAAGCAA	GTTGACAGATCCGGTGACCT
*TaHMA4*	GATGAAGAGCAGCCTGGTG	CCGTGAAAGGGAACAGGAC
*TaHMA5*	CAACATCATCGGCATCCCG	TAGTACCTCAGCAGGAGG
*TaHMA6*	AACATCGTTGCCATCCCTGT	TTTCTATATCTCCTCAGCAGCAG
*TaHMA7*	CGTCGATAGCTGGAGCACTA	CTCCGTCTGACACACCAGAA
*TaHMA8*	ATGGCGAAAGTTCACCAGAA	GCCATCAGTCCTCCTGAAAG
*TaHMA9*	CTACTTCTTCGCCATGGCGTA	TCACGGACGAGAAGGCCAT

### Database screening for sequence retrieval

Nine *Oryza sativa* HMA (OsHMA1-9) previously reported by Zhiguo et al. (2018) were retrieved from MSU Rice Genome Annotation Project Database (http://rice.uga.edu/)^14,26^ and crossed checked with the ARAMEMNON plant membrane database (http://aramemnon.uni-koeln.de/)^53^. These amino acid sequences were then used to retrieve putative HMA amino acid sequences of *Brachypodium distachyon (BdHAM1-9)* using Ensembl Plants (https://plants.ensembl.org/index.html)^54^. Information like Ensembl accession numbers, amino acid length, genomic length, and percentage similarity was also noted. HMAs of the remaining species (*Hordeum vulgare, Oryza brachyantha, Oryza barthii, Sorghum bicolor, Steria italica, Saccharum spontaneum, Triticum aestivum, Triticum urartu,* and *Zea mays)* were retrieved from Ensembl Plants through BLAST (Additional file S5). The Multiple Expectation Maximization for Motif Elucidation (MEME) webserver (https://meme-suite.org/meme/tools/meme)^55^ was used to determine HMA conserved motifs. These motifs were DKTGT, HP CPx/SPC, TGE, and GDxxNDxP, as previously reported by Fang et al. (2016)^20^. The distribution type of motifs was set as zero (0) or ‘one occurrence per sequence’ (zoops). The number of motifs and the minimum and maximum widths were set as 6, 15, and 50, respectively.

### Multiple sequence alignment (MSA) and phylogenetic analysis

MSA of all amino acids was performed using the web server NGPhylogen.fr (https://ngphylogeny.fr/)^56^. A Maximum Likelihood (MLH) tree was constructed using advanced workflow. MAFFT for MSA^57^, BMGE for MSA file trimming^58^, and Fast-Tree for phylogenetic MLH tree with 1000 bootstrap value were selected^59^^,^^60^. The resulting tree was visualized and refined using the iTOL online tool (http://itol.embl.de/index.shtml)^61^.

### Physiochemical properties, gene structure and domain identification in TaHMA1-27

Sequence information was obtained for TaHMA1-27 from the Uniprot database, while chromosomal location and genomic length were from Ensembl Plants (https://www.uniprot.org/uniprot/^62^, https://plants.ensembl.org/index.html^63^). Other physiochemical properties like protein mass in Daltons (Da), hypothetical isoelectric point, and grand average of hydropathicity (GRAVY) were calculated using the ProtParam tool of ExPaSy (https://web.expasy.org/protparam/)^64^. The NCBI conserved domain database (CDD) (https://www.ncbi.nlm.nih.gov/Structure/cdd/wrpsb.cgi)^65^ with search option Pfam V33.1-18271 PSSMs (http://pfam.xfam.org/)^66^ was used for distribution analysis of HMA conserved protein domains. The resultant domains diagram was drawn using the TB tools software^52^. The composition and position of exon/introns in *TaHMA1-27* homologs were analyzed by comparing the CDS of *TaHMA1-27* homologs to its corresponding genomic sequences using the online tool Gene Structure Display Server (GSDS) (http://gsds.gao-lab.org/)^67^.

### Subcellular localization and transmembrane helixes prediction

The subcellular localization of HMAs was predicted using the WoLF PSORT protein localization predictor (https://wolfpsort.hgc.jp/)^68^. Resulting cellular signal values were used to construct a heatmap using the Tbtools software^52^. The TMHMM Server V. 2.0 (http://www.cbs.dtu.dk/services/TMHMM/)^69^ was used to predict the putative transmembrane helices of all TaHMA1-27 members.

### *TaHMA* chromosomal location, synonymous (Ka) and non-synonymous rates and synthetic relationship

Chromosomal location and synthetic relationship were illustrated using a phenoGram plot (http://visualization.ritchielab.psu.edu/phenograms/plot)^70^ and Circos v0.67^71^, respectively. The substitution rate of synonymous (Ks) and nonsynonymous (Ka) was calculated using the Ka/Ks calculator of TB tools software for the selective pressure determination^52^. The approximate divergence time of the duplication events for each paralogous gene pair a million years ago (MYA = 10^−6^) was determined using the formula T = Ks/2x*MYA where x is the mean synonymous substitution rate which is 6.56 × 10^−9^^72^.

### Ethics approval

Wheat seeds (var. Sehar-06) were collected from the seed bank of the National Agricultural Research Council (NARC), Islamabad. This variety is widely cultivated in Pakistan.

## Supplementary Information

Supplementary Information.

## Data Availability

The databases used in this research are open and publicly available. No administrative permissions are needed for accessing and using the data. The links to the databases are given below. Ensembl Plants (https://plants.ensembl.org/index.html). MEME webserver (https://meme-suite.org/meme/tools/meme). NGPhylogen.fr (https://ngphylogeny.fr/). iTOL online tool (http://itol.embl.de/index.shtml). Uniport database (https://www.uniprot.org/uniprot/). ProtParam tool of ExPaSy (https://web.expasy.org/protparam/). NCBI conserved domain database (CDD) (https://www.ncbi.nlm.nih.gov/Structure/cdd/wrpsb.cgi). Pfam V33.1-18271 PSSMs (http://pfam.xfam.org/). Gene Structure Display Server (GSDS) (http://gsds.gao-lab.org/). WoLF PSORT (https://wolfpsort.hgc.jp/). TMHMM Server V. 2.0 (http://www.cbs.dtu.dk/services/TMHMM/). Phenogram plot (http://visualization.ritchielab.psu.edu/phenograms/plot).
