# Supplementary material for: Genome-wide analysis of heavy metal ATPases (HMAs) in Poaceae species and their potential role against copper stress in Triticum aestivum
Source: Sci Rep. 2023 May 9;13:7551. doi: 10.1038/s41598-023-32023-7 (PMC10170112; doi:10.1038/s41598-023-32023-7)
Supplement: Supplementary file 1 — Supplementary Information. [file 41598_2023_32023_MOESM1_ESM.pdf]

Additional file 1: HMA Sequences retrieval details of studied host.

S. no	OsHMAsID	BdHMAsID	Description on uniprot	Uniprot ID	Mass	bp	aa	Data base	Percentage Identity
1.	OsHMA1 LOC_Os06g 47550.1	KQK17265	Uncharacterized protein	I1GWE5	87,509	2925	819	Ensemble Plants	91.7
2.	OsHMA2; Os06t0700700-02	KQK17390	HMA domain-containing protein	I1GWN6	111,708	4358	1039	Ensemble Plants	89.5
3.	OsHMA3; LOC_Os07g 12900.1	KQK20301	Putative cadmium/zinc-transporting P1B-ATPase 3 HMA3.1	A0A0C5QLA7	89,335	2982	819	Ensemble Plants	77.1
4.	OsHMA4; LOC_Os02g 10290.1	KQJ93861	Uncharacterized protein	I1HYC6	105,921	4255	918	Ensemble Plants	90.7
5.	OsHMA5; LOC_Os04g 46940.1	KQJ83984	Uncharacterized protein	A0A0Q3ID01	112,272	3614	1035	Ensemble Plants	90.6
6.	OsHMA6; LOC_Os02g 07630.1	KQJ93558	Uncharacterized protein	I1HXQ7	106,763	3444	996	Ensemble Plants	90.6
7.	OsHMA7; LOC_Os08g 37950.1	KQJ98742	HMA domain-containing protein	I1I827	100,112	3503	954	Ensemble Plants	90.0
8.	OsHMA8; LOC_Os03g 08070.1	KQK23343	HMA domain-containing protein	I1H931	93,202	3591	891	Ensemble Plants	88.1
9.	OsHMA9; LOC_Os06g 45500.1	KQK17019	Uncharacterized protein	I1GVX7	108,138	3039	1012	Ensemble Plants	89.4
	**BdHMAsID**	**Obarthii HMAsID**	**Description on uniprot**	**Uniprot ID**	**Mass**	**bp**	**aa**	**Data base**	**Percentage Identity**

1	KQK17265	OBART06G25490.1	Uncharacterized protein	A0A0D3GK65	87,277	2469	822	Ensemble Plants	91.8
2	KQK17390	OBART06G26400.1	HMA domain-containing protein	A0A0D3GKG3	116,246	3210	1069	Ensemble Plants	89.9
3	KQK20301	OBART07G08010.1	Uncharacterized protein	A0A0D3GNX3	80,725	2343	780	Ensemble Plants	90.4
4	KQJ93861	OBART02G07350.1	Uncharacterized protein	A0A0D3F1Z8	105,146	3455	978	Ensemble Plants	90.8
5	KQJ83984	OBART04G21220.1	Uncharacterized protein	A0A0D3FYS3	112,631	3576	1042	Ensemble Plants	90.6
6	KQJ93558	*OBART02G05500.1	Uncharacterized protein	A0A0D3F1B8	106,254	2982	993	Ensemble Plants	90.7
7	KQJ98742	OBART08G18640.1	Uncharacterized protein	A0A0D3H1J5	90,967	3187	862	Ensemble Plants	91.6
8	KQK23343	OBART03G05730.1	HMA domain-containing protein	A0A0D3FEJ7	95,116	2733	910	Ensemble Plants	90.5
9	KQK17019	*OBART02G05500.1	Uncharacterized protein	A0A0D3F1B8	106,254	2982	993	Ensemble Plants	86.8
	**BdHMAsID**	**ZmHMAs**	**Description on uniprot**	**Uniprot ID**	**Mass**	**bp**	**aa**	**Data base**	**Percentage Identity**
1	KQK17265	Zm00001eb226120_T001	Putative cadmium/zinc-transporting ATPase HMA1 chloroplastic	A0A1D6GU97	53,433	3195	832	Ensemble Plants	91.9

2	KQK17390	Zm00001eb226870_T001	Cadmium/zinc-transporting ATPase HMA2	A0A1D6GV05	107,086	4156	1241	Ensemble Plants	86.9
3	KQK20301	Zm00001eb095010_T003	Cadmium/zinc-transporting ATPase HMA2	A0A1D6EKZ4	99,575	3215	894	Ensemble Plants	74.2
4	KQJ93861	Zm00001eb236270_T001	Putative copper-transporting ATPase HMA5	A0A1D6H478	105,973	3608	974	Ensemble Plants	90.7
5	KQJ83984	Zm00001eb074360_T001	Putative ATP dependent copper transporter	A0A1D6E3L5	112,100	3673	1036	Ensemble Plants	86.2
6	KQJ93558	Zm00001eb207090_T001	Copper-transporting ATPase RAN1	A0A1D6QS85	74,187	3512	998	Ensemble Plants	87.7
7	KQJ98742	Zm00001eb199150_T001	Copper-transporting ATPase PAA1 chloroplastic	A0A1D6QK32	70,458	3306	928	Ensemble Plants	88.0
8	KQK23343	Zm00001eb005820_T003	Copper-transporting ATPase PAA2 chloroplastic	A0A1D6JQC9	84,826	3227	916	Ensemble Plants	88.0
9	KQK17019	Zm00001eb389830_T001	Copper-transporting ATPase RAN1	A0A1D6P5L6	109,213	3755	1002	Ensemble Plants	89.0
	**BdHMAsID**	**SbHMAsID**	**Description on uniprot**	**Uniprot ID**	**Mass**	**bp**	**aa**	**Data base**	**Percentage Identity**

1	KQK17265	KXG20697	Uncharacterized protein	A0A194YL74	87,947	2867	828	Ensemble Plants	92.4
2	KQK17390	EER96099	HMA domain-containing protein	C5XDI2	91,794	3007	895	Ensemble Plants	66.3
3	KQK20301	EER96100	HMA domain-containing protein	C5XDI3	96,262	3122	933	Ensemble Plants	77.0
4	KQJ93861	EES04697	Uncharacterized protein	C5XXH4	105,223	4290	974	Ensemble Plants	90.7
5	KQJ83984	EES11247	Putative copper-exporting ATPase	Q6JAG2	108,736	3419	1002	Ensemble Plants	89.8
6	KQJ93558	EES06348	Uncharacterized protein	C5XW52	107,150	3147	997	Ensemble Plants	100
7	KQJ98742	OQU81022	HMA domain-containing protein	A0A1Z5RB93	83,093	3554	787	Ensemble Plants	94.4
8	KQK23343	KXG40056	HMA domain-containing protein	A0A1B6QQ54	94,401	3280	900	Ensemble Plants	91.4
9	KQK17019	KXG20553	Uncharacterized protein	A0A194YKS2	106,891	3345	1007	Ensemble Plants	90.2
	**BdHMAsID**	**TuHMAsID**	**Description on uniprot**	**Uniprot ID**	**Mass**	**bp**	**aa**	**Data base**	**Percentage Identity**
1	KQK17265	TRIUR3_22875-T1	Putative cadmium/zinc-transporting ATPase HMA1, chloroplastic	M8AA02	76,781	2157	718	Ensemble Plants	98.0

2	KQK17390	TRIUR3_09526-T1	Cadmium/zinc-transporting ATPase 3	M7Z923	103,439	2907	968	Ensemble Plants	100
3	KQK20301	TRIUR3_18572-T1	Cadmium/zinc-transporting ATPase 3	M7Z1B1	62,683	1997	592	Ensemble Plants	92.8
4	KQJ93861	TRIUR3_12628-T1	Putative copper-transporting ATPase 3	M7ZEH4	105,623	2943	980	Ensemble Plants	93.1
5	KQJ83984	TRIUR3_07892-T1	Putative copper-transporting ATPase 3	M7Z1T4	98,309	2706	901	Ensemble Plants	94.1
6	KQJ93558	TRIUR3_31446-T1	Copper-transporting ATPase RAN1	M7Y9I2	102,994	2853	950	Ensemble Plants	95.0
7	KQJ98742	*TRIUR3_12613-T1	Putative copper-transporting ATPase PAA1	M7ZW28	105,261	2922	973	Ensemble Plants	94.7
8	KQK23343	*TRIUR3_12613-T1	Putative copper-transporting ATPase PAA1	M7ZW28	105,261	2922	973	Ensemble Plants	50.4
9	KQK17019	TRIUR3_03709-T1	Copper-transporting ATPase RAN1	M7YJH0	101,531	2838	945	Ensemble Plants	95.2

	**BdHMAsID**	**SsHMAsID**	**bp**	**aa**	**Database**	**Percentage Identity**
1	KQK17265	Sspon.08G0003060-2P-mRNA-1	2762	772	Ensemble Plants	93.0
2	*KQK17390	Sspon.02G0022050-1A-mRNA-1	3063	903	Ensemble Plants	69.1

3	*KQK20301	Sspon.02G0022050-1A	3063	903	Ensemble Plants	80.4
4	KQJ93861	Sspon.04G0015780-2D-mRNA-1	2883	960	Ensemble Plants	90.3
5	KQJ83984	Sspon.05G0024030-1P-mRNA-1	2988	995	Ensemble Plants	87.8
6	*KQJ93558	Sspon.04G0017330-1P-mRNA-1	3045	999	Ensemble Plants	83.6
7	KQJ98742	Sspon.06G0000460-2C-mRNA-1	2781	956	Ensemble Plants	88.0
8	KQK23343	Sspon.01G0004310-2C-mRNA-1	3250	894	Ensemble Plants	90.0
9	*KQK17019	Sspon.04G0017330-1P-mRNA-1	3045	999	Ensemble Plants	88.5

	**BdHMAsID**	**HvHMAsID**	**Description on uniprot**	**Uniprot ID**	**Mass**	**bp**	**aa**	**Data base**	**Percentage Identity**
1	KQK17265	HORVU7Hr1G100160.2	Uncharacterized protein	A0A287XKH5	92,585	3129	871	Ensemble Plants	93.0
2	KQK17390	HORVU7Hr1G097210.4	HMA domain-containing protein	M0WLW4	100,371	3600	946	Ensemble Plants	86.6
3	KQK20301	HORVU5Hr1G094430.8	HMA domain-containing protein	A0A287SBM8	80,512	2784	765	Ensemble Plants	81.5
4	KQJ93861	HORVU6Hr1G033380.2	Uncharacterized protein	A0A287TV87	106,113	3208	987	Ensemble Plants	93.2
5	KQJ83984	HORVU2Hr1G097010.3	Uncharacterized protein	A0A287J245	73,112	3850	672	Ensemble Plants	93.9
6	*KQJ93558	HORVU7Hr1G108890.1	Uncharacterized protein	A0A287XS00	109,572	3203	1023	Ensemble Plants	85.4

7	*KQJ98742	HORVU7Hr1G 108890.1	Uncharacter ized protein	A0A287X S00	109,572	32 03	102 3	Ense mble Plants	38.6
8	KQK23343	HORVU4Hr1G 076330.4	Uncharacter ized protein	A0A287P QG1	65,573	24 55	621	Ense mble Plants	91.6
9	*KQK17019	HORVU7Hr1G 108890.1	Uncharacter ized protein	A0A287X S00	109,572	32 03	102 3	Ense mble Plants	93.2
	**BdHMAsID**	**ObrachyHMAs ID**	**Description on uniprot**	**Uniprot ID**	**Mass**	**bp**	**aa**	**Data base**	**Percen tage Identit y**
1	KQK17265	OB06G33300.1	Uncharacter ized protein	J3MH36	88,408	27 10	831	Ense mble Plants	91.8
2	KQK17390	OB06G34010.1	HMA domain-containing protein	J3MHA7	113,166	35 43	104 4	Ense mble Plants	90.4
3	KQK20301	OB07G15960.1	HMA domain-containing protein	J3MJL4	94,038	27 09	902	Ense mble Plants	77.9
4	KQJ93861	OB02G16630.1	Uncharacter ized protein	J3LAJ6	105,103	29 31	976	Ense mble Plants	90.0
5	KQJ83984	OB04G28260.1	Uncharacter ized protein	J3M0A1	107,518	30 04	999	Ense mble Plants	89.8
6	*KQJ93558	OB02G14740.1	Uncharacter ized protein	J3LA07	97,756	35 57	904	Ense mble Plants	89.4
7	KQJ98742	OB08G25750.1	HMA domain-containing protein	J3MTZ4	85,691	29 86	807	Ense mble Plants	88.0
8	KQK23343	OB03G15820.1	Uncharacter ized protein	J3LKK6	77,012	26 08	728	Ense mble Plants	91.5
9	*KQK17019	OB02G14740.1	Uncharacter ized protein	J3LA07	Uncharact erized protein	35 57	904	Ense mble Plants	85.8

	BdHMAsID	SiMAsID	Description on uniprot	Uniprot ID	Mass	bp	aa	Data base	Percentage Identity
1	KQK17265	KQL11874	Uncharacterized protein	K3XVA3	88,235	3929	828	Ensemble Plants	91.6
2	KQK17390	KQL11767	HMA domain-containing protein	K3XUY1	118,938	3721	1095	Ensemble Plants	87.5
3	KQK20301	KQL23074	HMA domain-containing protein	K4A2L2	84,426	2415	805	Ensemble Plants	81.4
4	KQJ93861	KQL28281	Uncharacterized protein	K3YPN3	104,882	3827	974	Ensemble Plants	90.3
5	KQJ83984	KQK98407	Uncharacterized protein	K3Y4W9	107,974	3141	999	Ensemble Plants	88.2
6	KQJ93558	KQL11044	Uncharacterized protein	K3XV11	107,318	3345	1007	Ensemble Plants	85.7
7	KQJ98742	KQL02276	HMA domain-containing protein	K3YG30	100,563	3433	963	Ensemble Plants	89.3
8	KQK23343	KQK92321	HMA domain-containing protein	K4A5J1	94,510	3134	903	Ensemble Plants	90.6
9	KQK17019	KQL28533	Uncharacterized protein	K3YPL7	106,165	3106	993	Ensemble Plants	86.6

*Accessions which were repeated and excluded during tree refining.

Additional file 2 (A): Conserved motifs sequences and width of *TaHMA1-27* analyzed by MEME webserver

Motifs #	Sequence	Width
**1**	TGVGASRGVLIKGGDVLERLANVDAIVFDKTGTLTKGKPVV	41
**2**	KVLPGEKVPVDGVVVWGQSHVBESMJTGESAPVAKZVGSEVIGGTVNLBG	50
**3**	RQNYVWALAYNIVAIPVAAGALFPFTGLRLPPWLAGACMAFSSVSVVCSS	50
**4**	QKEGGPVAMVGDGINDAPALAAADVGMAM	29
**5**	SALAQIIRLVEEAQMSKAPVQRLADKVAGYF	31
**6**	SVLVIACPCALGLATPTAVMV	21
**7**	IGRRFYVAAYRALKHGSPNMDVLVALGTTAAYVYSVV	37
**8**	GFVAPKFFEESAMJISFVLLGKYLEVLAKGKASDAMSKLMELAPETAVLL	50
**9**	GLISVSDPJKREAAEVISELKSMGISSVMLTGDNWEAA	38
**10**	EAADVVLMKNBLEDVITAIDLSRKTFRRI	29
**11**	LVASAEANSEHPLAKAIVEYAQSFH	25
**12**	QKILSCRLDIGGMTCASCVNSVERILKKLPGVKRAAVALATELAEVEYDP	50
**13**	DFEALPGKGVVAEIDGKLVLVGNKRLMAE	29
**14**	TWFLCGTLGAYPNSWIPETSBSFVLALQF	29
**15**	KGVGJEEVRAELKPEDKAEKV	21

Additional file 2 (B): Conserved motif analysis of *TaHMA1-27* proteins using MEME webserver. Conserved motifs are present with their respective clades of phylogenetic tree in rectangular form. Color gradient represents bootstrap value. Motifs 1, 10, 11, 13, 14 and 15 are present in all *TaHMAs*, whereas motif 9 is present in clade 2 and 3 only.

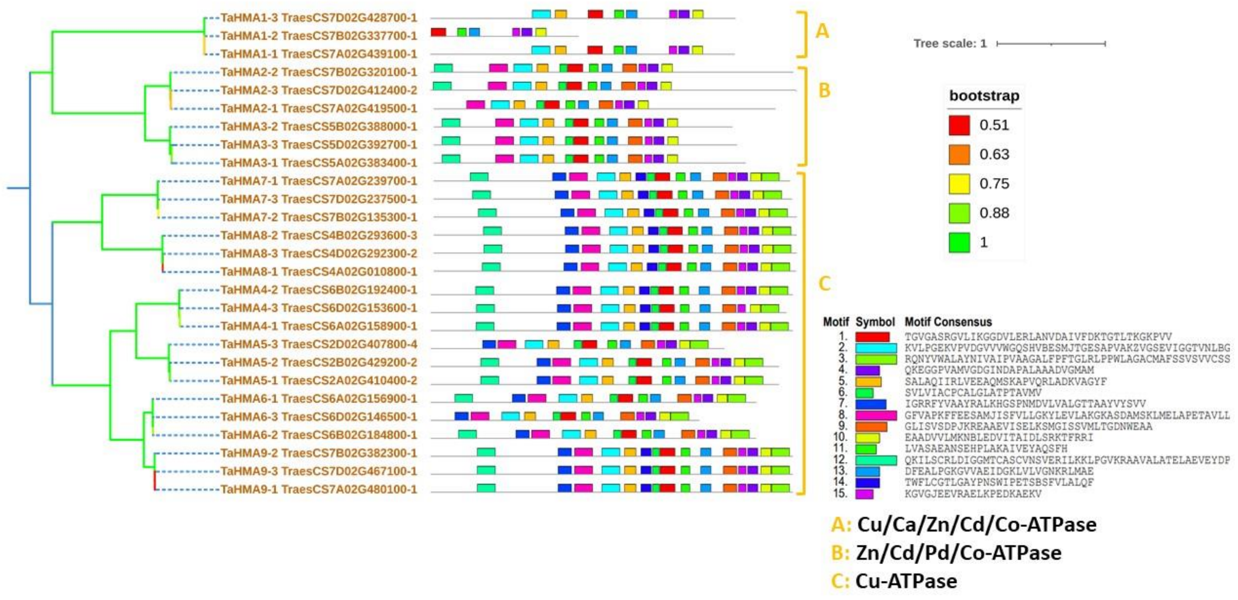

Additional file 3: Gene structure of *TaHMAs*. The exons are indicated in orange shade whereas introns are indicated in black. The length of the genes can be estimated using the scale given.

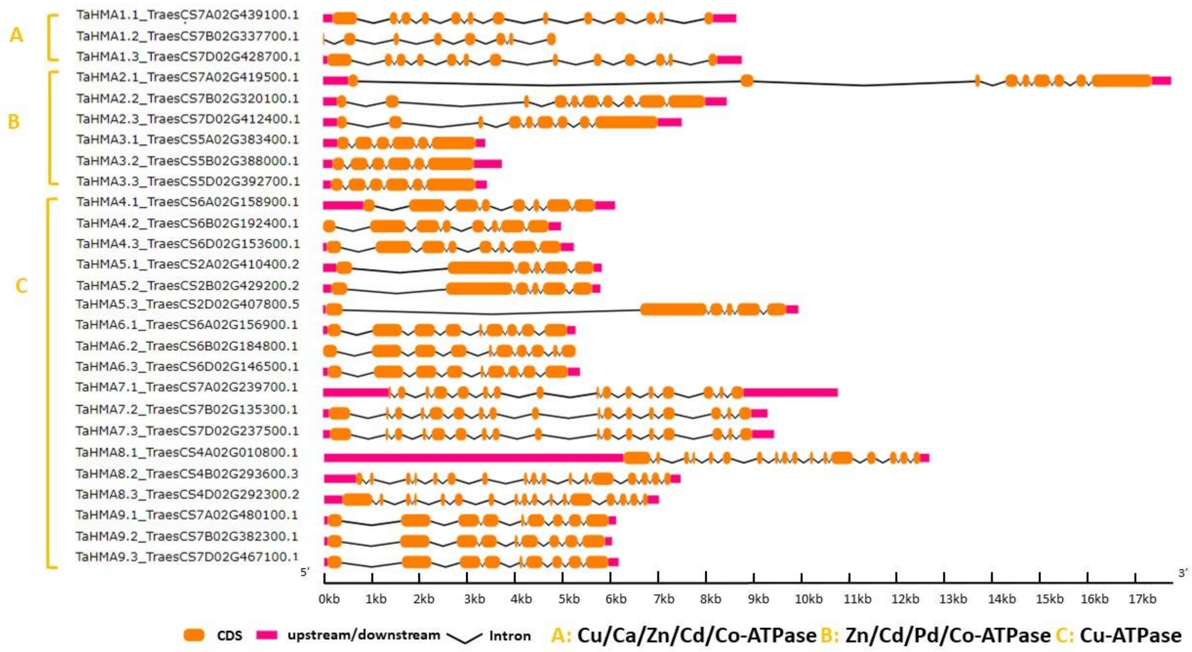

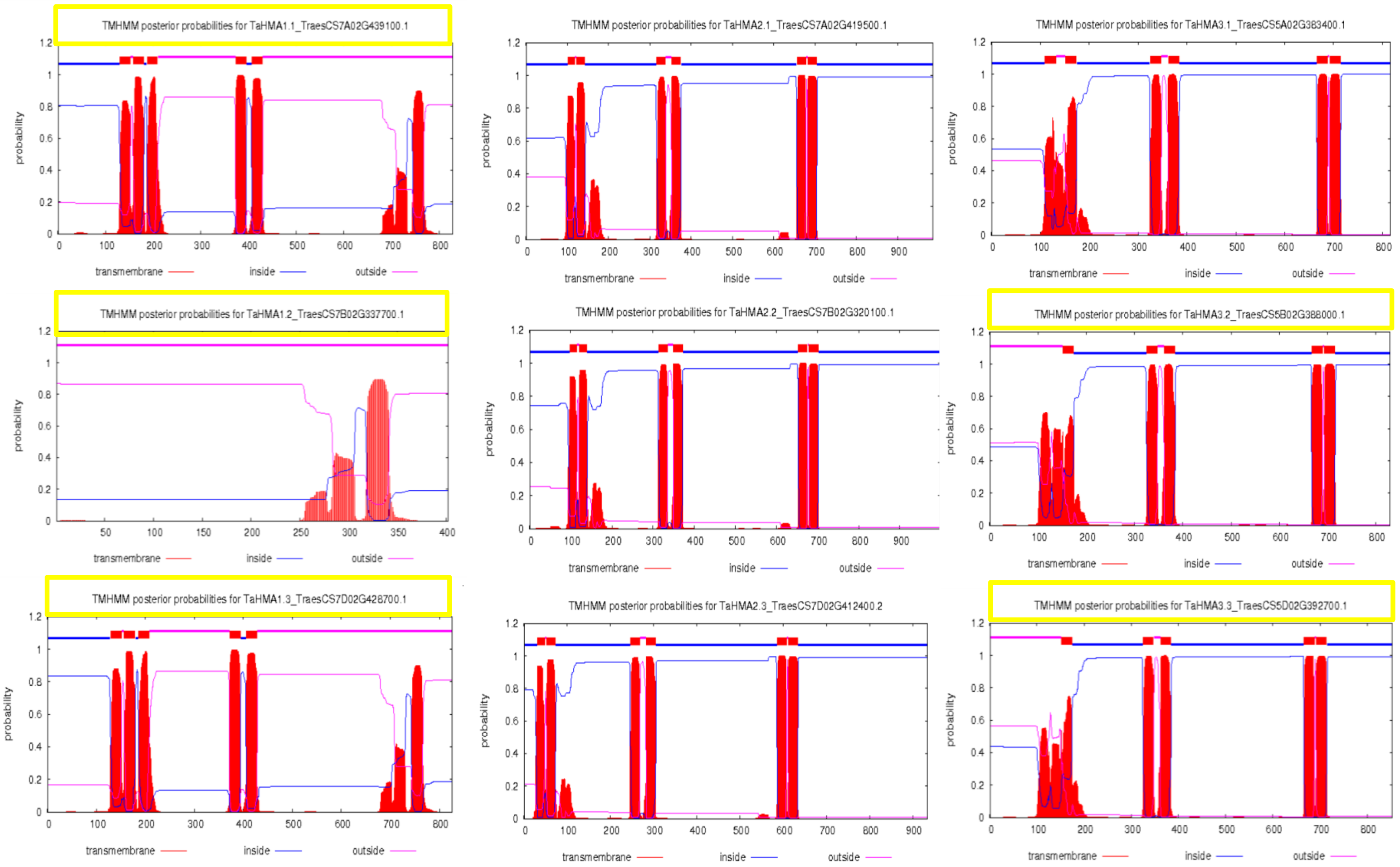

Additional file 4 (A): TMH analysis of *TaHMA1-3*. Yellow box is representing protein in which transmembrane helices are less than six.

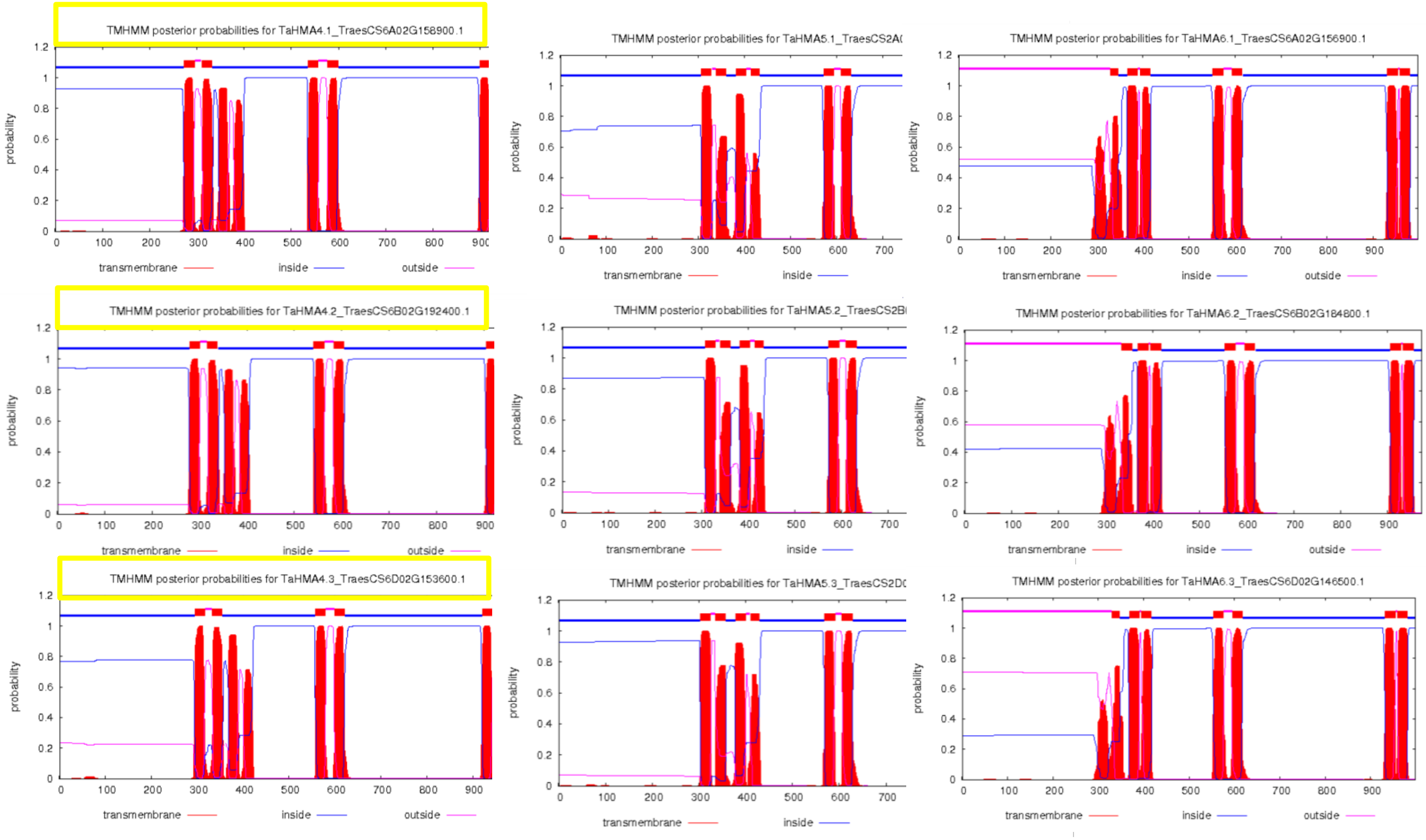

Additional file 4 (B): TMH analysis of *TaHMA4-6*. Yellow box is representing protein in which transmembrane helices are less than six

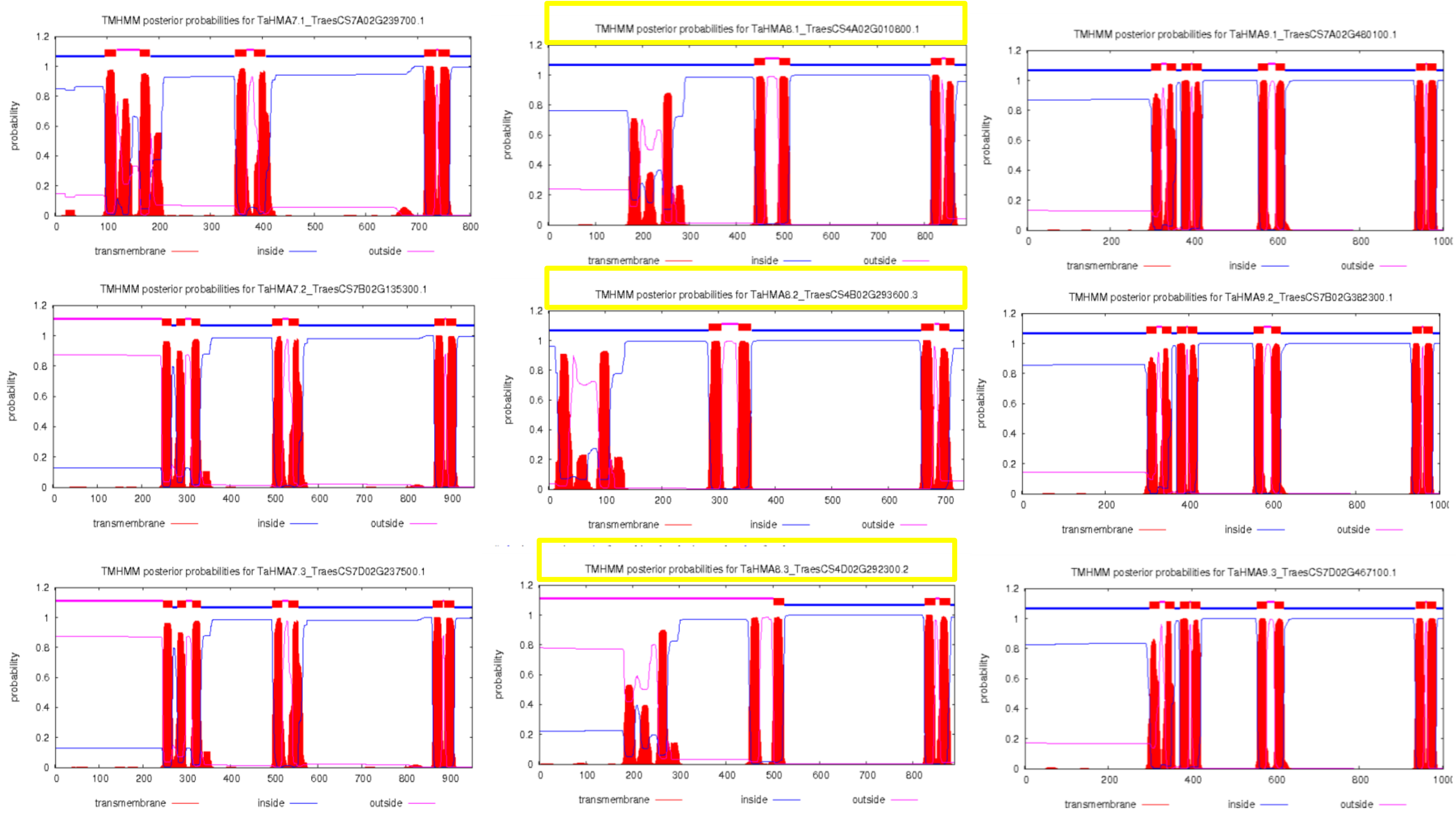

Additional file 4 (C): TMH analysis of *TaHMA7-9*. Yellow box is representing protein in which transmembrane helices are less than six

Additional file 5: All retrieved sequences of *TaHMAs1-27* with accession numbers, gene names, chromosome number, location start-end, Strand, CDS length, protein length, mass in dalton, pI, GRAVY, no. of exon/intron, uniport description and genomic length.

Transcript ID	TaHMA 1-27	Chromosome	Location Start-End	Strand	CDS (bp)	Length (aa)	Protein Moelcular Weight (Da)	pI	GRAVY	No.of Exon	No.of Inrton	Genomic Length
TraesCS7A02G439100.1	TaHMA 1.1	7A	634,378,971 - 634,387,635	F	2487	828	88,131	6.81	0.18	13	12	8.66
TraesCS7B02G337700.1	TaHMA 1.2	7B	592,506,452 - 592,511,328	F	1209	402	42,548	6.37	0.25	8	7	4.88
TraesCS7D02G428700.1	TaHMA 1.3	7D	548,885,379 - 548,894,155	F	3729	826	87,769	6.99	0.177	13	12	8.78
TraesCS7A02G419500.1	TaHMA 2.1	7A	611,316,717 - 611,334,498	F	2958	985	106,456	6.29	-0.162	9	8	17.78
TraesCS7B02G320100.1	TaHMA 2.2	7B	570,450,770 - 570,459,233	F	2997	998	107,407	6.51	-0.087	10	9	8.46
TraesCS7D02G412400.2	TaHMA 2.3	7D	530,907,556 - 530,915,075	F	3012	934	101,121	6.59	-0.152	8	7	6.48
TraesCS5A02G383400.1	TaHMA 3.1	5A	581,137,113 - 581,140,512	F	2562	816	85,658	6.29	0.279	6	5	3.4
TraesCS5B02G388000.1	TaHMA 3.2	5B	567,018,067 - 567,021,814	F	2490	829	86,817	6.05	0.293	6	5	3.75
TraesCS5D02G392700.1	TaHMA 3.3	5D	460,941,086 - 460,944,519	F	2451	853	89,091	6.12	0.277	6	5	3.43
TraesCS6A02G158900.1	TaHMA 4.1	6A	150,964,243 - 150,970,363	F	2925	974	105,106	5.44	0.169	8	7	6.12

TraesCS6B02G192400.1	TaHMA 4.2	6B	226,399,885 - 226,404,875	F	2991	980	105,627	5.44	0.174	8	7	4.99
TraesCS6D02G153600.1	TaHMA 4.3	6D	127,382,802 - 127,388,058	F	2943	996	107,410	5.44	0.185	8	7	5.26
TraesCS2A02G410400.2	TaHMA 5.1	2A	668,067,544 - 668,073,388	F	2982	993	107,089	5.62	0.205	6	5	5.84
TraesCS2B02G429200.2	TaHMA 5.2	2B	616,494,297 - 616,500,111	F	2985	994	107,491	5.88	0.181	6	5	5.82
TraesCS2D02G407800.4	TaHMA 5.3	2D	522,990,194- 523,000,156	F	3003	990	107,092	6.04	0.192	6	5	5.62
TraesCS6A02G156900.1	TaHMA 6.1	6A	146,000,586 - 146,005,878	F	2994	997	106,854	5.23	0.325	9	8	5.29
TraesCS6B02G184800.1	TaHMA 6.2	6B	207,923,884- 207,929,181	F	2919	972	104,694	5.53	0.291	10	9	5.3
TraesCS6D02G146500.1	TaHMA 6.3	6D	117,414,359 - 117,419,743	F	2997	998	107,150	5.39	0.297	9	8	5.38
TraesCS7A02G239700.1	TaHMA 7.1	7A	214,311,509 - 214,322,302	R	2412	803	85,188	6.28	0.161	16	15	10.79
TraesCS7B02G135300.1	TaHMA 7.2	7B	165,301,384 - 165,310,703	R	2859	952	100,020	7.23	0.114	17	16	9.32

TraesCS7D02G 237500.1	TaHMA 7.3	7D	201,883,715 - 201,893,169	R	2897	952	99,938	6.9 8	0.123	17	16	9.46
TraesCS4A02G 010800.1	TaHMA 8.1	4A	6,020,338- 6,033,023	F	2673	890	93,266	5.8 6	0.161	18	17	12.69
TraesCS4B02G 293600.3	TaHMA 8.2	4B	579,391,172 - 579,398,644	R	2205	734	77,098	5.4 7	0.218	17	16	7.47
TraesCS4D02G 292300.2	TaHMA 8.3	4D	463,402,923 - 463,409,941	R	2670	889	92,774	6	0.208	17	16	7.02
TraesCS7A02G 480100.1	TaHMA 9.1	7A	672,069,735 - 672,075,854	F	3006	100 1	106,890	5.2 9	0.308	9	8	6.12
TraesCS7B02G 382300.1	TaHMA 9.2	7B	648,103,932 - 648,109,965	F	3006	100 1	106,766	5.2 8	0.311	9	8	6.03
TraesCS7D02G 467100.1	TaHMA 9.3	7D	580,570,22 8- 580,576,400 f	F	3006	100 1	106,780	5.4	0.323	9	8	6.17
